# Supplementary figures and images for: Characterization of two thermophilic cellulases from Talaromyces leycettanus JCM12802 and their synergistic action on cellulose hydrolysis
Source: PLoS One. 2019 Nov 15;14(11):e0224803. doi: 10.1371/journal.pone.0224803 (PMC6857856; doi:10.1371/journal.pone.0224803)

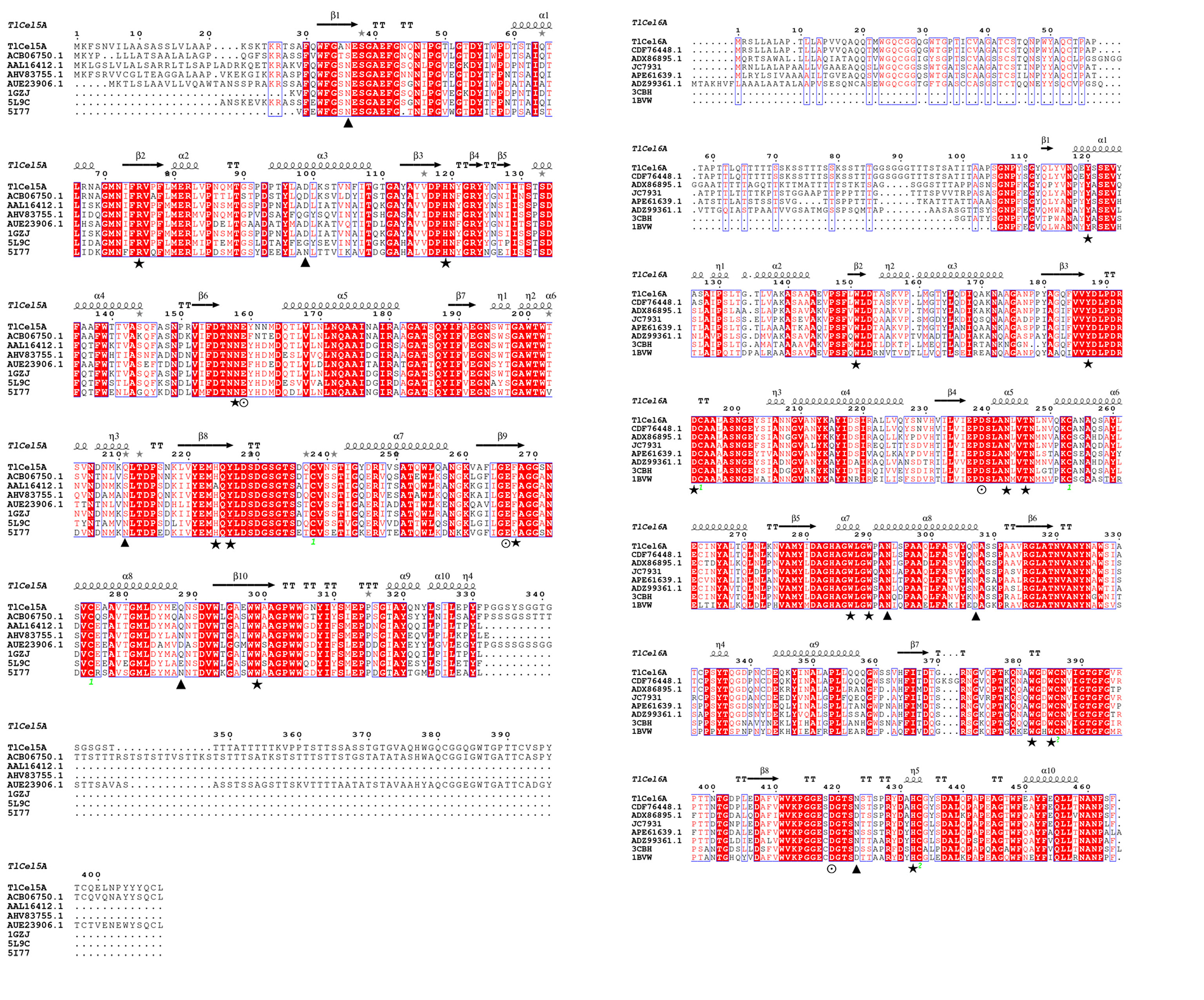

Supplement: S1 Fig — Multiple sequence alignment of TlCel5A (A) and TlCel6A (B) with known sequences. The catalytic residues are indicated by circles. The conserved residues are indicated with asterisks. And the potential N-glycosylation sites are indicated by triangles. (TIF) [file pone.0224803.s001.tif]

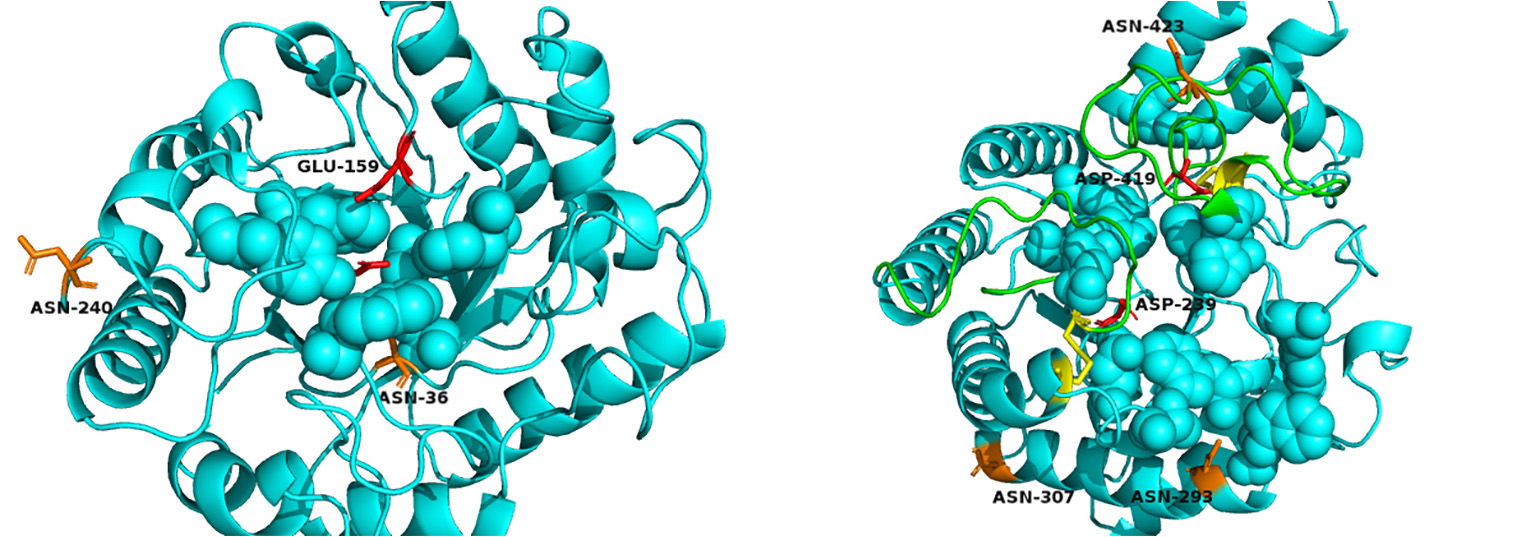

Supplement: S2 Fig — Modeled structures of TlCel5A (A) and TlCel6A (B). Orange sticks represent potential N-glycosylation sites, red sticks indicate the catalytic residues, and spheres indicate the conserved residues. (TIF) [file pone.0224803.s002.tif]

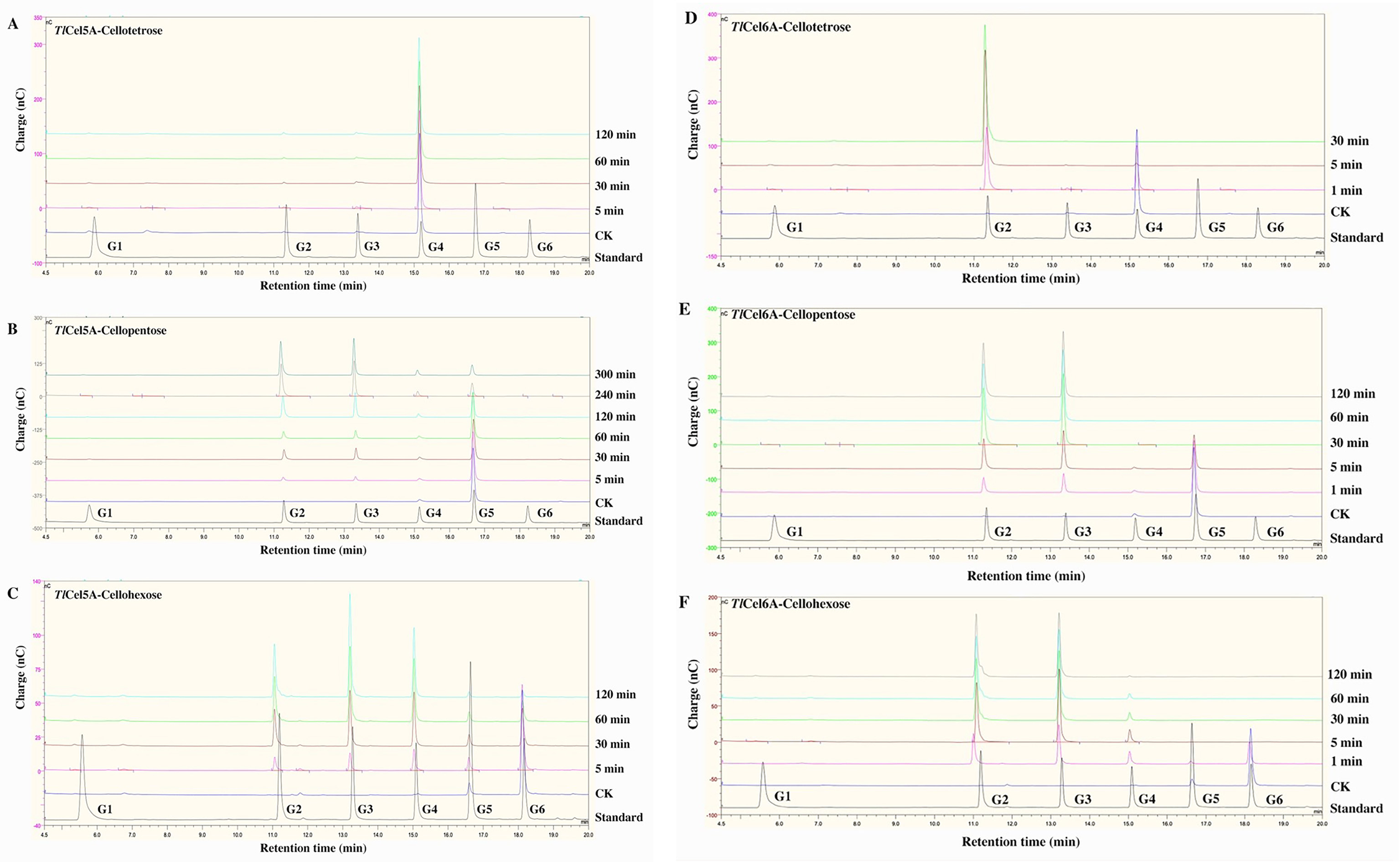

Supplement: S3 Fig — (TIF) [file pone.0224803.s003.tif]

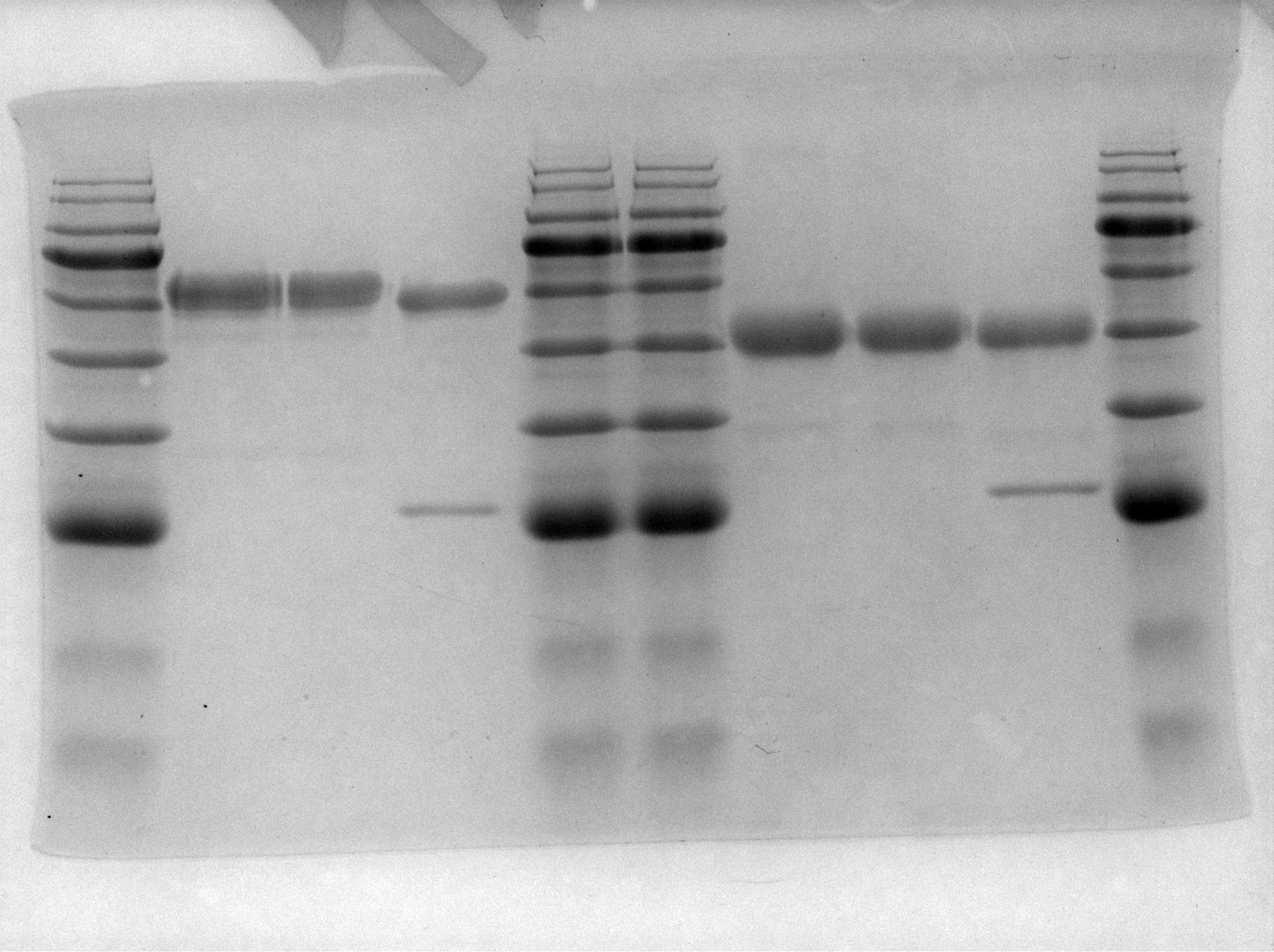

Supplement: S1 Raw Images — (TIF) [file pone.0224803.s004.tif]
