# Supplementary material for: Characterization of two thermophilic cellulases from Talaromyces leycettanus JCM12802 and their synergistic action on cellulose hydrolysis
Source: PLoS One. 2019 Nov 15;14(11):e0224803. doi: 10.1371/journal.pone.0224803 (PMC6857856; doi:10.1371/journal.pone.0224803)
Supplement: Attachment — Submitted filename: Response to reviewers.docx [file pone.0224803.s005.docx]

Key Laboratory for Feed Biotechnology of the Ministry of Agriculture and Rural Affairs

Feed Research Institute

Chinese Academy of Agricultural Sciences

Beijing 100081, P. R. China

Email: luohuiying@caas.cn

Oct. 19, 2019

To the Editorial Office of PLOS ONE

Dear Professor Jean-Guy Berrin,

We are resubmitting our manuscript PONE-D-19-21437 entitled “**Characterization of two thermophilic** **cellulases from** ***Talaromyces leycettanus* JCM12802 and their synergistic action on cellulose hydrolysis**” for a potential publication in PLOS ONE. The manuscript has undergone significant revisions according to the reviewers’ comments. All revisions are marked blue in the revised manuscript. The following are our replies to the Reviewers’ comments.

Your kind consideration is much appreciated.

With kind regards,

Huiying Luo

**Response to editor:**

1. Please ensure that your manuscript meets PLOS ONE's style requirements, including those for file naming.

Answer: We have checked the manuscript carefully and changed the name of my files to ensure meet PLOS ONE's style requirements.

2. PLOS ONE now requires that authors provide the original uncropped and unadjusted images underlying all blot or gel results reported in a submission’s figures or Supporting Information files.

Answer: I have provided the original uncropped and unadjusted gel image in the Supporting Information file.

3. In your cover letter, please note whether your blot/gel image data are in Supporting Information or posted at a public data repository.

Answer: The gel image data has been provided in the Supporting Information file.

4. Figures are of poor quality and need to be replaced.

Answer: The figures with high quality have been replaced and uploaded.

5. To enhance the reproducibility of your results, we recommend that if applicable you deposit your laboratory protocols in protocols.io, where a protocol can be assigned its own identifier (DOI) such that it can be cited independently in the future.

Answer: I have finished the protocol and uploaded it, this is the link: dx.doi.org/10.17504/protocols.io.8g4htyw.

**Response to Reviewer 1:**

Reviewer #1: Several of the references are old and should be replaced or supplemented with more recent publications

Answer: The latest references have been added and inserted into the corresponding position in the article and marked yellow. The followings are the six newly inserted references.

3. Touijer H, Benchemsi N, Ettayebi M, Janati A, Chaouni B, Bekkari H. Thermostable Cellulases from the Yeast *Trichosporon sp*. Enzyme research. 2019;1-6. (Line42)

6. Kamal S, Khan SU, Muhammad N, Shoaib M, Omar M, Kaneza P, et al. Insights on heterologous expression of fungal cellulases in *Pichia pastoris*. Biochemistry and Molecular Biology. 2018; 3(1): 15-35. (Line47)

15. Kamal S, Khan SU, Khan S, Shoaib M, Khan H, Man S, et al. Recent view on heterologous expression of thermostable fungal cellulases, focused on expression factory of *Pichia Pastoris*. International Journal of Basic Medical Sciences and Pharmacy. 2018; 7(2), 43-57. (Line66)

21. Li D C, Papageorgiou A C. Cellulases from thermophilic fungi: recent insights and biotechnological potential. Enzyme Research. 2019; 395-417. (Line72)

22. Xu X, Fan C, Song L, Li J, Chen Y, Zhang Y, et al. A novel CreA-mediated regulation mechanism of cellulase expression in the thermophilic fungus *Humicola* *insolens*. International Journal of Molecular Sciences. 2019; 20(15): 3693. (Line72)

30. Fang H, Zhao R, Li CF, Zhao C. Simultaneous enhancement of the beta–exo synergism and exo–exo synergism in *Trichoderma reesei* cellulase to increase the cellulose degrading capability. Microbial Cell Factories. 2019; 18(1): 9-23. (Line103)

**Response to Reviewer 2:**

Reviewer #2: I recommend this manuscript for publication in PLOS ONE. I suggest the following issue to address though. Speaking about the synergistic action, the Authors should make some thoughts and provide chemical scheme of hydrolysis reaction pathway for this scenario. It will simplify understanding the concept of enzymes action.

Answer: We would like to take this opportunity to thank Reviewer 2 for his/her suggestions on my paper with kindly words. And the chemical scheme of hydrolysis reaction pathway of synergistic action was as follows:

“Cooperation between many functionally related proteins is very important in biology. A prominent example is the apparent rate of substrate conversion increased by the addition of different combination cellulase enzymes to break down cellulose. A high level of coordination between the enzyme, e.g. exo/endo, exo/exo and endo/the BG synergistic effect, which is effective to hydrolyze crystalline cellulose required. [1] [2] [3] [4]

There are three significant types of cellulases to be used basically for the efficient degradation of cellulose. And perhaps the most widely known mechanism remains the so-called endo/exo synergy model. In this form, cellobiohydrolases (CBHs), also called exo-glucanases, cleave cellobiose from the reducing and nonreducing ends producing crystalline ends of cellulose; Endo-glucanases (EGs) break recurrently the glycosidic bonds inside the amorphous part of the substrate creating new attack sites for the CBHs. The products of cellobiohydrolases and endoglucanases are able to inhibit the activities of their own enzymes, so finally, beta-glucosidases (BGLs) hydrolyze unconfined cellobiose and various soluble cellodextrins into glucose monomers. The efficient hydrolysis of cellulose requires the composition of a complete and balanced cellulase, with all components of the cellulase co-acting the enzymatic hydrolysis of cellulose. Synergy is used to describe the ability of collaboration between enzymes. [4] [5] [6] [7] [8]

For my experiments, the enzymes of *Tl*Cel5A and *Tl*Cel6A were combined at different ratios, and the commercial β-glucosidase from *Aspergillus fumigatu* (Sigma-Aldrich) was added at the concentration of 10% (mol β-glucosidase/mol total enzyme) to hydrolyze cellobiose to glucose finally. When using Avicel, phosphoric acid swollen cellulose (PASC) or steam-exploded corn straw (SECS) as the substrate, combination of *Tl*Cel5A and *Tl*Cel6A showed significant synergistic action, releasing more reduced sugars than the individual enzymes. (Line270)

1. Shang B Z, Chu J W. Kinetic modeling at single-molecule resolution elucidates the mechanisms of cellulase synergy. ACS Catalysis. 2014; 4(7): 2216-2225.

2. Van Dyk J S, Pletschke B I. A review of lignocellulose bioconversion using enzymatic hydrolysis and synergistic cooperation between enzymes-factors affecting enzymes, conversion and synergy. Biotechnology Advances. 2012; 30(6):1458-1480.

3. Yang B, Dai Z, Shi-You Ding, et al. Enzymatic hydrolysis of cellulosic biomass. Biofuels. 2011; 2(4):421-450.

4. Kamal S, Khan S U, Muhammad N, et al. Insights on heterologous expression of fungal cellulases in *Pichia pastoris*. Biochemistry and Molecular Biology. 2018; 3(1): 1-15.

5. Olsen J P, Borch K, Westh P. Endo/exo-synergism of cellulases increases with substrate conversion. Biotechnology and bioengineering. 2017; 114(3): 696-700.

6. Badino S F, Christensen S J, Kari J, et al. Exo-exo synergy between Cel6A and Cel7A from *Hypocrea jecorina*: Role of carbohydrate binding module and the endo-lytic character of the enzymes. Biotechnology and bioengineering. 2017; 114(8): 1639-1647.

7. Boisset C, Fraschini C, Schülein M, et al. Imaging the enzymatic digestion of bacterial cellulose ribbons reveals the endo character of the cellobiohydrolase Cel6A from *Humicola* *insolens* and its mode of synergy with cellobiohydrolase Cel7A. Applied & Environmental Microbiology. 2000; 66(4): 1444-1452.

8. Fang H, Zhao R, Li C, et al. Simultaneous enhancement of the beta-exo synergism and exo-exo synergism in *Trichoderma reesei* cellulase to increase the cellulose degrading capability. Microbial cell factories. 2019; 18(1): 1-9.
